# Neuromuscular efficiency in schoolchildren: a 16-week structured exercise program improves plantar load distribution across static, dynamic and load-bearing tasks

**DOI:** 10.7717/peerj.21941

**Published:** 2026-11-12

**Authors:** Aida Bendo, Denis Nuriu

**Affiliations:** 1 Department of Movement and Health, Faculty of Physical Activity and Recreation, Sports University of Tirana, Tirana, Albania; 2 Department of Physical Activity and Health, Institute of Scientific Research in Sport, Sports University of Tirana, Tirana, Albania

**Keywords:** Gait efficiency, Neuromuscular adaptation, Plantar pressure, Postural control, Schoolchildren, Structured exercise

## Abstract

**Background:**

Neuromuscular efficiency in children is associated with the control of postural and gait mechanics, which are dependent on physical activity levels. Alterations in plantar pressure distribution provide objective indices of load control and efficiency of movement under various functional conditions. The study aimed to explore the effects of a 16-week structured exercise program on plantar pressure distribution and neuromuscular efficiency in schoolchildren at static, dynamic, and load-bearing conditions.

**Methods:**

For a total of 147 schoolchildren, a controlled experimental design where pre–post measurements were conducted (experimental: *n* = 74; control: *n* = 73). Plantar pressure was evaluated during standing, ambulation, and walking with a schoolbag. Statistical analyses were conducted with paired and independent t-tests, and two-way mixed analysis of variance (ANOVA).

**Results:**

There was a significant reduction in plantar pressure in the experimental condition (standing: *p* = .024; walking: *p* = .017; load: *p* = .010), while there were no significant changes in the control group. Weight-bearing symmetry increased appreciably for the experimental group (*p* < .001). Group and condition effects were found to have significant main effects in ANOVA, alongside significant interaction effects (*p* < .05), showing that changes are felt more under the conditions of dynamic or load-bearing tasks.

**Conclusions:**

The results show that systematic exercise increases neuromuscular utilization by optimizing distribution of loads, postural coordination and functional adaptation. The gradual improvement across all training conditions indicates that the greatest improvements occurred under dynamic and load-bearing conditions compared with quiet standing. These findings also support the development of a structured model of exercise at school for the promotion of postural health and optimal function of movement in children.

## Introduction

Gait and posture are recognized as key indicators of neuromuscular and musculoskeletal development in children, reflecting the integrated function of the nervous, muscular, and skeletal systems. These roles are involved in balance, movement efficiency, and functional capacity in adult development (Bach, Daffertshofer & Dominici, 2021). Gait should represent a complicated sensorimotor process and represent more than a mechanical activity which relies on somatosensory, vestibular, and visual information to support dynamic balance and adaptive movements in developing individuals (Miyagishima et al., 2023). Further, the proprioception of posture relies on coordinated activation of stabilising musculature to establish the body aligned against gravity, and to avoid mechanical load on musculoskeletal structures (Salsali et al., 2023). Regular physical activity (PA) plays a crucial role in supporting these processes by enhancing neuromuscular coordination, maintaining muscular strength, and promoting joint stability. Studies show that structured exercise interventions improve vital spatiotemporal gait parameters such as stride length, cadence and walking speed and mitigate asymmetry and energetic cost (Agostini et al., 2015). Consistently, balance and core stability programs, focusing on postural control among school-aged children, have shown dramatic improvements (Ruivo, Carita & Pezarat-Correia, 2016). On the other hand, lower doses of PA and increased sedentary habits have been linked to decreased muscular function and reduced postural regulation, with less efficient movement patterns, which in turn can lead to eventual musculoskeletal dysfunction in children (Guo, He & Wang, 2025). Gait and posture maturation is a complex developmental process including both structural and neural changes. This is due to growth-related changes in limb length, muscle mass, and tendon stiffness that are able to improve mechanical leverage and force transmission, and neural maturation, including myelination of corticospinal pathways increases motor precision and coordination (Froehle et al., 2012; Galloway, Lalley & Shearn, 2013). Locomotor control progresses from the predominantly reflexive regulation to the refined cortical and subcortical regulation that includes the cerebellum and basal ganglia involved in timing and coordination and flexibility of movement (Dominici et al., 2010; Martínez-Romero, De Ste Croix & Sainz de Baranda, 2025). This is seen in the improvements achieved in gait symmetry, reduced variability, and increased movement efficiency (Hausdorff, 2005; Miyagishima et al., 2023). Successful postural control also relies on the simultaneous reception of sensory information from the visual, vestibular, and proprioceptive systems that help control the position of the center of mass throughout static and dynamic movements (Mani et al., 2021). This integration of the sensorimotor system constitutes the foundation of efficient neuromuscular control, enabling coordinated movement, postural stability, and optimal load distribution throughout the musculoskeletal system. Yet, despite these adaptive responses, evidence from epidemiology reveals that gait and postural deviance is widespread in school-aged children, up to 30% to 65% (Martínez-Romero, De Ste Croix & Sainz de Baranda, 2025). Such changes are often linked with sedentary lifestyle, obesity and rapid growth phases, causing biomechanical imbalances, compensatory modes of movement, and more localized loading (Salsali et al., 2023; Horsak et al., 2019). Unchecked, these deviations may evolve into enduring musculoskeletal disorders; the potential for the delay detection and treatment has therefore arisen. Recent developments in biomechanical assessment, including assessment of plantar pressure, have brought us the ability to objectively and sensitively assess distribution of force, stability and gait symmetry. These methods allow for the estimation of subtle asymmetries and inefficiencies that could not be reported *via* clinical measurements in isolation (Cousins, Morrison & Drechsler, 2013; Keatsamarn et al., 2021). Moreover, school-based screening with such technologies has been successful in early identification of deviations and prevention intervention guided by an organized PA program (Liu et al., 2025). The present intervention was designed as a progressive, school-based, multi-component exercise program in accordance with current recommendations for children and adolescents, incorporating balance, coordination, core stabilization, and lower-limb strengthening exercises through age-appropriate supervised activities (Behm et al., 2008; Bull et al., 2020; Faigenbaum et al., 2009). A 16-week intervention period was selected because previous school-based studies have demonstrated that programs lasting approximately 12–16 weeks provide sufficient time to induce meaningful improvements in muscular fitness, postural control, functional movement, and lower-limb biomechanics in school-aged children (Cox et al., 2020; Wu et al., 2023; Zhu et al., 2025). Whilst previous studies have documented the beneficial effects of PA on isolated components of motor function, most have evaluated balance, gait, or postural control separately, without simultaneously examining plantar pressure distribution, weight-bearing symmetry, and functional performance across static standing, dynamic walking, and load-bearing conditions. Consequently, it remains unclear whether exercise-induced neuromuscular adaptations are consistent across different functional tasks and mechanical demands. The load-bearing condition was included because carrying a schoolbag represents one of the most common functional tasks performed daily by school-aged children. This activity imposes additional mechanical and postural demands that may alter plantar pressure distribution, weight-bearing symmetry, and postural control, making it clinically relevant for evaluating the functional effects of exercise interventions. This comprehensive approach is anticipated to provide a better understanding of adaptive neuromuscular mechanisms and support evidence-based strategies for improving postural health and movement efficiency in pediatric populations. Hence, the objective of the present study was to evaluate the effects of a 16-week structured exercise program on neuromuscular control in schoolchildren by examining changes in plantar pressure distribution, postural control, weight-bearing symmetry, and movement efficiency under static, dynamic, and load-bearing conditions.

We hypothesized that participants in the experimental group would demonstrate greater improvements in plantar pressure distribution, postural control, weight-bearing symmetry, and movement efficiency than those in the control group following the 16-week intervention. Furthermore, we expected these improvements to be evident across static standing, dynamic walking, and load-bearing conditions, with the greatest changes observed during the more functionally demanding tasks.

## Methods

### Study design

In this study design, students completed a controlled experimental protocol and followed a series of measures to examine the impact of a programme of exercise on neuromuscular efficiency in school children. Pre-post analysis was utilized in this study, so that changes over time in each group could be assessed, and a controlled pre–post intervention design was used to compare an experimental group receiving a structured exercise program with a control group that continued the regular physical education curriculum. The design was designed to assess plantar load distribution by functional conditions such as static standing, dynamic walking, and load-bearing locomotion.

### Participants

The study included 147 school-aged children, with 74 participants allocated to the experimental group and 73 to the control group. All participants were recruited from primary schools and were at a developmental stage characterized by ongoing neuromuscular maturation. Children were eligible if they were healthy, able to walk independently without assistive devices, and met the predefined inclusion criteria. Exclusion criteria included diagnosed neurological or musculoskeletal disorders, recent lower-limb injuries, structural abnormalities affecting gait, and participation in specialized training programs outside the regular school curriculum. Baseline anthropometric characteristics of the participants, including age, height, weight, body mass index (BMI), and sex distribution, are presented in Table 1.

### Sampling procedure

Participant recruitment followed a multistage sampling procedure. In the first stage, five cities from different geographical regions of Albania were randomly selected to ensure broad geographical representation of the study sample. In the second stage, one primary school was randomly selected from each selected city. In the third stage, all children within the predefined age range were screened for eligibility according to the inclusion and exclusion criteria. Participant selection was based on eligibility criteria regardless of sex. Children were excluded if they did not meet the inclusion criteria, met any exclusion criterion, were absent during data collection, lacked written parental consent, withdrew from the study, or did not complete all study assessments.

### Group allocation

Following participant recruitment, the participating schools were allocated to the study groups according to the logistical feasibility of implementing the structured exercise program. Three schools with the necessary facilities, infrastructure, and qualified personnel to deliver the intervention were assigned to the experimental group, whereas the remaining two schools continued their regular physical education curriculum and served as the control group. Consequently, all eligible children within each participating school received the intervention corresponding to their school’s allocation.

### Sample size and power analysis

A sensitivity power analysis was conducted using G*Power software (version 3.1) to evaluate the adequacy of the final sample size for the primary between-group comparison of pre-to-post change scores. The analysis was based on a two-tailed independent-samples *t*-test, an alpha level of 0.05, a statistical power of 0.80, and the final sample of 147 participants, comprising 74 participants in the experimental group and 73 in the control group. The analysis indicated that the available sample was sufficient to detect a minimum standardized between-group effect size of Cohen’s *d* = 0.47, corresponding to a small-to-moderate effect. Thus, the final sample provided adequate statistical sensitivity for the primary between-group analyses.

**Table 1:** Baseline characteristics of the study participants.

**Variable**	**Experimental (*n* = 74)**	**Control (*n* = 73)**	***p*** **-value**
Age (years)	12.58 ± 1.05	13.12 ± 1.79	0.027
Height (cm)	161.22 ± 8.71	162.30 ± 10.02	0.485
Weight (kg)	53.34 ± 11.26	58.27 ± 13.21	0.016
BMI (kg/m^2^)	20.46 ± 3.89	21.99 ± 4.08	0.022
Male, n (%)	35 (47.3)	29 (39.7)	0.448
Female, n (%)	39 (52.7)	44 (60.3)	–
Standing plantar pressure (Pre, N/cm^2^)	13.50 ± 6.33	12.71 ± 6.25	0.446
Walking plantar pressure (Pre, N/cm^2^)	52.41 ± 13.50	51.67 ± 12.47	0.730
Walking with schoolbag plantar pressure (Pre, N/cm^2^)	54.18 ± 11.84	57.09 ± 9.42	0.101

**Notes.**

Values are presented as mean ± standard deviation (SD), unless otherwise indicated. Group comparisons were performed using independent-samples t-tests for continuous variables and the chi-square test for sex distribution.

BMI body mass index Pre pre-intervention

### Ethical considerations

The study was performed in accordance with the stated ethical guidelines for research with human subjects as per the Declaration of Helsinki. Ethics approval: This study was approved by the Research Ethical Council of the University of Sports of Tirana (Approval Protocol No. 915/2; approved on 14 May 2024). Written informed consent was obtained from parents or guardians of the participants prior to participation, and assent was obtained from all children. Study participants were briefed on the study procedures and the right to withdraw from the study at any stage without any negative consequences.

### Intervention program

The experimental participants engaged in a 16-week exercise program from September 2 to December 20, 2024, to improve neuromuscular function and postural control. The program included exercises aimed at core stability, balance, coordination, and lower-limb strength, increasing in complexity in accordance to the participants’ developmental level. Training sessions were implemented two to three times per week during school hours. Each session lasted about 45 to 60 min; and the control group continued to participate in the regular school physical education curriculum, without receiving the additional structured exercise intervention administered to the experimental group. A detailed week-by-week description of the structured exercise program, including exercise content, sets, repetitions, session duration, and progression criteria, is provided in Table S1. During the functional integration phase, schoolbags or external loads were individualized and did not exceed 10% of each participant’s body mass.

All exercise sessions were supervised by certified physical education teachers to ensure correct exercise execution and participant safety. Exercise progression followed the predefined 16-week protocol (Table S1), with progressive increases in exercise complexity, postural control demands, balance challenges, and functional task integration across the four intervention phases. Participants were required to attend at least 80% of the scheduled sessions to be included in the final analysis. Only participants who completed both the intervention and the pre- and post-intervention assessments were included in the statistical analyses.

### Instrumentation

The plantar pressure reading achieved was accomplished using the T&T Medilogic^®^ System (T&T Medilogic Medizintechnik GmbH, Schönefeld, Germany), a high-resolution pressure distribution platform on the plantar surface. The system permits accurate determination of pressure magnitude expressed as N/cm^2^ and load distribution across the entire process in either stationary or dynamic space. The apparatus in bioengineering is well-established in biomechanical research, and in the field of pediatric health it offers a reliable and reproducible means to assess behavior in children. In addition to plantar pressure magnitude (N/cm^2^), the T&T Medilogic^®^ System automatically provides the percentage distribution of body weight between the left and right feet. Weight-bearing symmetry was assessed directly from this bilateral load distribution generated by the system software under each testing condition. No additional derived symmetry index or mathematical formula was applied.

### Testing procedures

All measurements were performed under three standard conditions to obtain a response to various functional demands. Participants in the stationary condition were upright, standing without shoes, with a natural stance and forward vision. To avoid changes in the dynamic conditions, participants walked at a self-selected comfortable speed, at a pace they could control while they were walking on the measuring platform to create natural gait patterns, while walking in each experiment. Participants in the load-bearing condition progressed to the walking trials, carrying a typical schoolbag used to represent the standard daily load during each step of the exercise and performing these steps in walking exercises, as intended. All evaluations were carried out before and after the 16-week intervention under the same testing conditions for comparison purposes for all participants to prevent variation. The assessor responsible for collecting plantar pressure measurements was blinded to participants’ group allocation throughout both the pre- and post-intervention assessments.

### Outcome measures

The main outcome variable was maximum plantar pressure (N/cm^2^) as an indicator of localised load concentration as well as load distribution effectiveness. This parameter is characterized not only by stability in standing position in static circumstances but also the biomechanical performance in gait. Also, weight-bearing symmetry (%) was examined as a secondary outcome, characterized by the proportional distribution of load between the left and right limbs and that reflects postural alignment as well as neuromuscular coordination.

### Statistical analysis

Within-group and between-group comparisons were carried out statistically. All variables were measured with descriptive statistics (means and standard deviation). Within groups, paired-samples t-tests were employed to determine groups after the treatment; independent-samples t-tests were used to compare the mean change score between the experimental and control groups. We used a two-way mixed analysis of variance (ANOVA) (Group × Time) to investigate the effect of group and functional condition together, examining both main and interaction effects of the dependent conditions of standing, walking, and load-bearing conditions. Effect sizes were reported by Cohen’s d for paired comparisons and by partial eta squared (*η*^2^p) for ANOVA results. Statistical significance was assumed at *p* < .05, providing solid support for the interpretation of intervention effects in neuromuscular efficiency and plantar load distribution. To control the family-wise Type I error rate associated with multiple pairwise comparisons across the three functional conditions, Bonferroni correction was applied to the primary between-group comparisons. For these three comparisons, statistical significance was evaluated using an adjusted alpha level of 0.0167 (0.05/3). All statistical analyses were performed using IBM SPSS Statistics, version 27.0 (IBM Corp., Armonk, NY, USA), whereas the sensitivity power analysis was conducted using G*Power, version 3.1.

## Results

Key results are presented in Tables 1–3 and Figs. 1 and 2, while additional detailed descriptive statistics and supporting graphical representations are provided in the Supplementary Materials (Tables S1–S5 and Figs. S1–S3).

Baseline participant characteristics and baseline plantar pressure measurements are presented in Table 1. The experimental and control groups differed significantly in age (*p* = 0.027), body weight (*p* = 0.016), and BMI (*p* = 0.022), whereas no significant differences were observed for height (*p* = 0.485), sex distribution (*p* = 0.448), or baseline plantar pressure under the standing, walking, and walking-with-schoolbag conditions (all *p* > 0.05).

Analysis of plantar pressure distribution showed consistent improvements in the experimental group across all functional conditions following the 16-week structured exercise intervention. By comparison, the control group showed only slight and non-significant alterations. In general, reductions in maximum plantar pressure were observed in static standing, dynamic walking, and load-bearing conditions; this demonstrated increased efficiency in load distribution and neuromuscular control. These alterations were supported by increases in weight-bearing symmetry, indicating improved postural alignment and inter-limb coordination. This change was most notable in response to task complexity, and there was a positive effect on the task load. Taken together, these results suggest that the intervention not only improved static postural control, but functional adaptation under dynamic and mechanical load conditions too.

### Static condition (standing)

The descriptive statistics for maximum plantar pressure during quiet standing are presented in Table S2. The experimental group experienced significantly less plantar pressure after the intervention (*p* = .024), while the control group shows no statistically significant change (*p* = .40). The mean decrease in the experimental group (−1.52 N/cm^2^) was larger than that in the control group (−0.60 N/cm^2^), indicating a better distribution of static load. Between-group comparison did not meet the Bonferroni-adjusted significance threshold (t(145) = 2.11, *p* = .036, *η*^2^p = 0.031).

**Table 2:** Summary of changes across conditions by group.

**Condition**	**Control group** **Δ (Post–Pre)**	**Effect size**	**Experimental group Δ (Post–Pre)**	**Effect size**
Standing	–0.60	–0.10	–1.52	–0.27
Walking	–0.56	–0.08	–1.58	–0.28
Load	–0.69	–0.11	–1.78	–0.31

**Table 3:** Two-way mixed ANOVA results (Group × Time).

**Effect**	**F**	***p*-value**	***η*^2^ p**
Group	5.94	.016^*^	0.039
Time	9.83	<.001^**^	0.063
Group × Time	4.12	.018^*^	0.028

**Notes.**

* *p* < .05 statistically significant.

** *p* < .001 statistically very significant.

**Figure 1:**
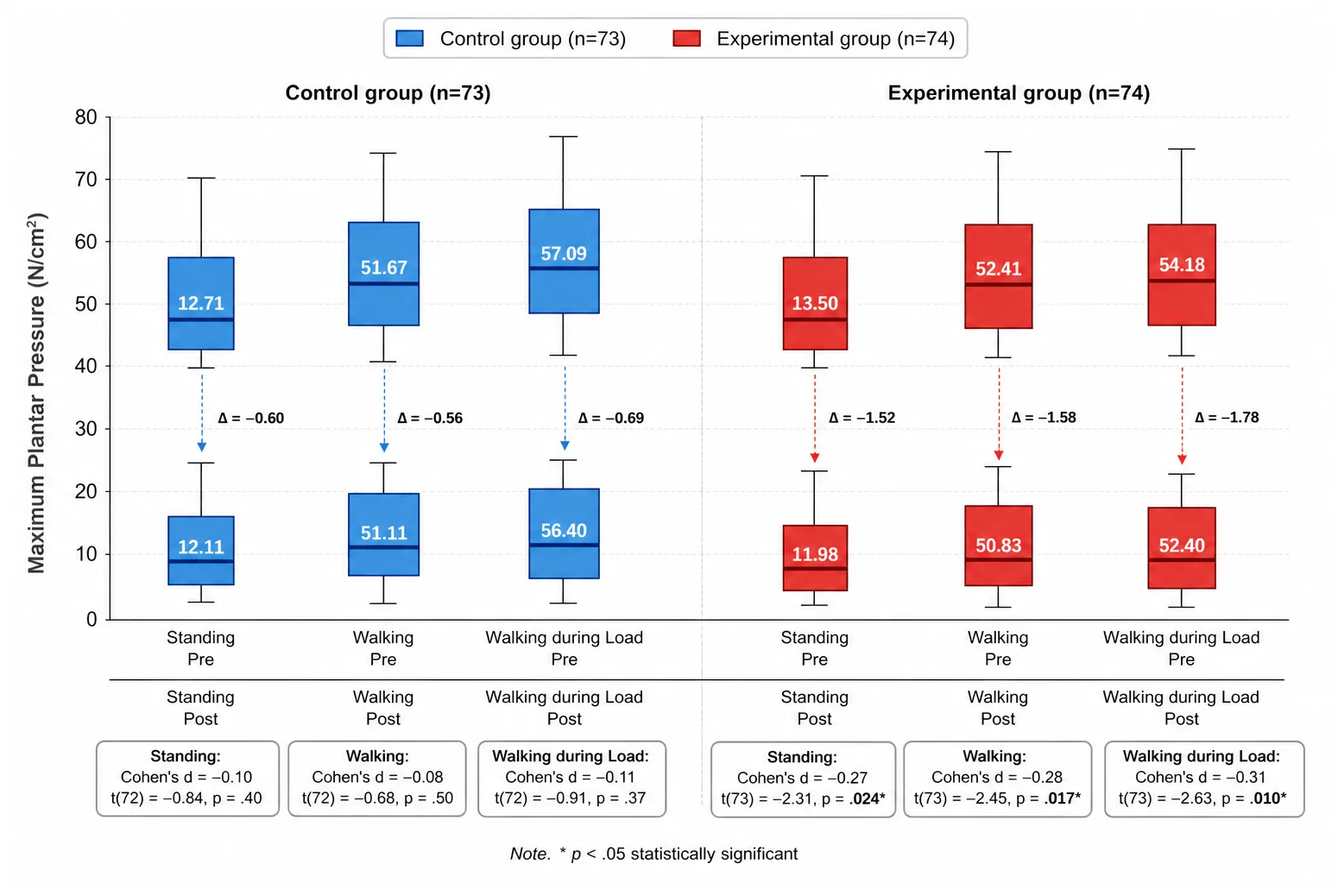
Comparison in plantar pressure among different functional conditions (boxplot).

**Figure 2:**
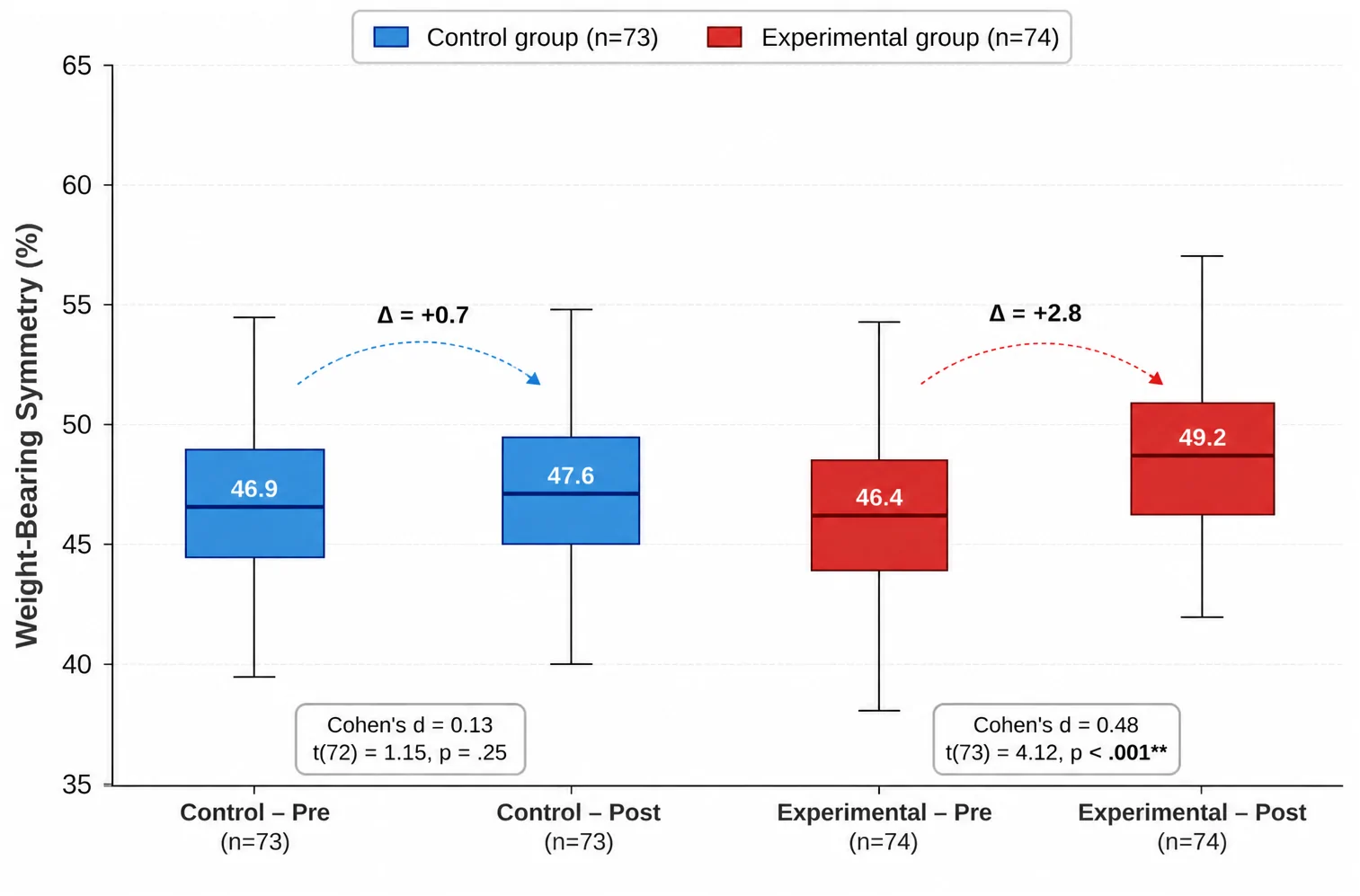
Weight-bearing symmetry (%): pre–post comparison (boxplot).

In this experiment, a significant reduction in median values was observed in postural load efficiency, as shown in Fig. S1. The changes were accompanied by a marginal reduction in the interquartile range, indicating a more consistent distribution of plantar pressure. Additionally, the pre- and pos*t*-test measurements in the control group showed little difference, indicating limited adaptation over time. Overall, the findings indicate improved redistribution of mechanical load during standing in the experimental group.

### Dynamic condition (walking)

The reduction in the experimental group (−1.58 N/cm^2^) is indicative of modified gait mechanics and a better transfer of loads during a walk, as shown in Table S3. The *p*-value was similar in between-group comparison,and did not meet the Bonferroni-adjusted significance threshold (t(145) = 2.27, *p* = .025, *η*^2^p = 0.034).

The greater reduction observed in the experimental group indicates more efficient gait mechanics and lower peak loading, as shown in Fig. S2. This reduction was accompanied by a slight decrease in the interquartile range, indicating a more uniform distribution of plantar pressure during walking. In contrast, the pre- and pos*t*-test values in the control group showed only minimal differences, indicating limited adaptation over time. Overall, the findings suggest a more efficient redistribution of mechanical load during dynamic gait in the experimental group.

### Load-bearing condition (walking with schoolbag)

Results under load-bearing condition are summarised in Table S4. The experimental group showed a significant reduction in plantar pressure (*p* = .010), whereas the change in the control group was again not statistically significant (*p* = .37). The greater mean reduction observed in the experimental group (−1.78 N/cm^2^) suggests an improved ability to cope with external loading. The differences between groups remained significant after Bonferroni correction (t(145) = 2.45, *p* = .016, *η*^2^p = 0.039).

Figure S3 shows the greatest reduction in the experimental group, suggesting improved load handling and lower peak plantar stress during load carriage. This contrast is also manifested by a downward change in the interquartile range indicating a more uniform and coordinated distribution of plantar pressure underloading. In contrast, few differences were observed in the control group compared with baseline, confirming limited adaptation to the applied load. Hence the pattern in the experimental group indicates higher mechanical efficiency and effective force redistribution during the load-bearing gait. The experimental group also shows a significant reduction indicating better load-distribution efficiency. A comparative analysis of conditions.

Overall, plantar pressure progressively decreased across all conditions in the experimental group (Table 2), with the greatest improvement observed under load-bearing conditions. This pattern suggests that as the tasks become more complex, neuromuscular adaptability increases.

Figure 1 presents grouped boxplots of maximum plantar pressure (N/cm^2^) under three functional conditions: standing, walking, and walking under load, for the experimental and control groups before and after the intervention. Pre- and post-intervention measurements are presented side by side, facilitating both within-group and between-group comparisons. Median plantar pressure was found to decrease significantly in the experimental group for all conditions including walking and load-bearing tasks. These transformations imply better neuromuscular efficiency and a more compact distribution of the mechanical load. In contrast, the control group showed only minimal, non-significant changes over time, indicating limited adaptation. These results collectively emphasize the success of the intervention to improve postural control and attenuate peak plantar loading in across different functional conditions.

#### Two-way mixed ANOVA

Based on the results presented in Table 3, the two-way mixed ANOVA revealed significant main effects of Group and Time, as well as a significant Group × Time interaction, indicating that changes over time differed significantly between the experimental and control groups.

Bonferroni-adjusted *post hoc* comparisons were performed where appropriate. These analyses did not alter the interpretation of the significant Group × Time interaction; therefore, only the overall ANOVA results are presented.

### Weight-bearing symmetry

The results for weight-bearing symmetry are presented in Table S5. This was significantly better in the experimental group (*p* < .001) by +2.8%, signifying a transition toward more balanced bilateral load distribution. The control group showed no significant change (*p* = .25). Between-group comparison confirmed a robust intervention effect (t(145) = 3.78, *p* < .001, *η*^2^p = 0.090).

Figure 2 shows the weight-bearing symmetry distribution for the control and experimental groups before and after the intervention. The median values for the experimental group shifted toward greater weight-bearing symmetry, whereas only minimal changes were observed in the control group.

## Discussion

This study examined the extent to which a 16-week exercise program could improve plantar pressure distribution and neuromuscular efficiency in schoolchildren under static, dynamic, and load-bearing conditions. The results provide evidence that the experimental group improved in load distribution, postural symmetry, and adaptation capabilities compared to the control group. These findings are consistent with the assumption that organized sport promotes integrated neuromuscular adaptations that do not depend only on single motor activities but apply to many conditions in the body. In the static phase, the decreased maximum plantar pressure represents improved postural alignment and more efficient weight-bearing strategies. Although the magnitude of improvement was modest, the statistically significant reduction in peak plantar pressure suggests a more uniform load distribution across the plantar surface. While the present study was not designed to establish clinically meaningful thresholds, these changes may reflect favorable adaptations in postural control, intrinsic foot muscle activation, and core stabilization that could contribute to improved movement efficiency during daily activities. These findings are consistent with previous evidence showing that enhanced postural control reduces localized mechanical stress and improves balance efficiency (Araújo, Moreira & Carvalho, 2023; Shourie et al., 2024). Additionally, reduced static plantar pressure implies less demand on a compensatory sway strategy with increased stability of the center of mass. During walking, the reduction in plantar pressure was greater and more consistent with enhanced gait efficiency and coordination because of the intervention. Less loading on the lower extremities during walking implies better shock absorption and a smoother transition through the stance phase. These adjustments are probably due to an improved neuromuscular collaboration between proximal and distal segments, notably for core stability and lower-limb mechanics. Indeed, better gait efficiency is thought to result from less erratic load transfer in higher degree of symmetry and lower variability of plantar loading (Lemire et al., 2023). In children, the present findings complement previous evidence by demonstrating that structured exercise effectively optimizes these mechanisms. The greatest improvements were observed under load-bearing conditions, as participants in the experimental group had the largest reduction in plantar pressure. This finding demonstrates the potential functional resilience to increase with mechanical demand that this intervention would provide. The application of an external load directly affects postural control, altering the center of mass and increasing the ground reaction forces. This decrease in plantar pressure indicates a better response to compensatory measures, such as trunk alignment and displacement of forces across the lower extremities. This is especially relevant considering prior studies that link high schoolbag load to abnormal gait mechanics and risk for postural changes in children (Grobler & Kramer, 2023). The capacity to reduce load-induced pressure increases demonstrates enhanced neuromuscular efficiency and mechanical adaptability. Another important finding of this study was the significant improvement in weight-bearing symmetry, indicative of bilateral coordination and postural stability. A more balanced load distribution may reduce unilateral dominance and compensatory overloading for one limb, which is essential for avoiding long-term musculoskeletal imbalance. Balanced load patterns promote efficient force transfer and minimize damage to joints and soft tissue, ultimately helping preserve joint and soft tissue integrity. This result appears to be in line with the evidence that neuromuscular training has been successful in normalizing asymmetrical motion patterns and enhancing bilateral coordination for children, according to previous work on motor-mechanical coordination (Goliwas et al., 2015). Notably, a gradient of significant improvement between conditions was demonstrated in the results, with the smallest difference on static standing, and largest variations in load-bearing tasks. This pattern is a task-dependent adaptation, indicating that the intervention not only improved general baseline postural control but also promoted learning to control movement as mechanical complexity increased. The interaction between Group × Time is also a powerful indicator of this, with increased task demands leading to enhanced neuromuscular adaptations. These results are consistent with the notion of sensorimotor transfer, which is the idea that gains in basic motor skills can transfer to more complex tasks involving motor action. Biomechanically, the decreases in plantar pressure and in symmetry reflect increased mechanical efficiency and lower compensatory muscle activity. In such cases, adequate gait and proper posture can be attributed to the balanced transition of medial–lateral pressure and an optimal alignment of the kinetic chain, reducing stress on joints and soft tissues (Adouni et al., 2024). There is sample evidence that with the intervention, this optimization took place, both making the movement patterns more economical and energy efficient, during both static and dynamic tasks. The results of this study correspond with previous studies showing benefits in postural and load management from structured PA for children. For example, Janakiraman et al. (2017) found that balance and coordination training led to improved plantar pressure symmetry, whereas Grobler & Kramer (2023) discussed exercise’s impact on alleviating the negative biomechanical effects of backpack load. Likewise, more recent systematic evidence shows that neuromuscular training improves static and dynamic balance, due to greater proprioceptive control and muscle coordination enhancements (Jastifer, 2025). This study contributes to the literature by providing a multidimensional assessment across static, dynamic, and load-bearing conditions. This approach offers a broader perspective on neuromuscular adaptation. This study has several practical implications as well as theoretical contributions. The results imply that school-based exercise programs can help play a key preventive role in postural health and musculoskeletal health in children. These interventions might thus provide an opportunity to decrease overuse injuries and promote better physical development during crucial growth phases (especially during years of relative physical growth). Overall, the present study demonstrates that a structured 16-week exercise program resulted in improvements in neuromuscular performance in schoolchildren. These improvements were reflected in reductions in maximum plantar pressure and improved load symmetry across different functional conditions. These results emphasize the need to build targeted PA into the school curriculum for better postural control, movement mechanics, and long-term musculoskeletal wellbeing.

This study has several limitations that should be considered when interpreting the findings. First, the intervention effects were evaluated immediately after the 16-week program, and no long-term follow-up was conducted to determine whether the observed improvements were maintained over time. Second, although participants were recruited from five schools located in different geographical regions of Albania, the sample may not fully represent all school-aged children nationwide, which may limit the generalizability of the findings. Additionally, the experimental and control groups differed significantly at baseline in age, body weight, and BMI. Although baseline plantar pressure did not differ significantly between groups, these demographic differences may have influenced the magnitude of the observed between-group effects and should therefore be considered when interpreting the intervention findings. Additionally, although the load-bearing condition was standardized by limiting the schoolbag weight to no more than 10% of each participant’s body mass, the exact load carried by each participant was not recorded or analyzed. Consequently, the potential influence of individualized load differences on plantar pressure responses could not be examined and should be considered in future research. This limitation should be considered specifically when interpreting the findings from the load-bearing condition, because individual differences in the actual schoolbag load may have contributed to variability in plantar pressure responses. Finally, although the control group continued to participate in the regular school physical education curriculum throughout the study, the specific content and intensity of these activities were not documented, limiting direct comparison with the structured exercise intervention. Future studies should include longer follow-up periods, larger and more geographically diverse samples, the recording and analysis of individualized schoolbag loads, and a more detailed characterization of control group activities.

## Conclusions

The present study demonstrated that a 16-week structured exercise intervention produced significant improvements in neuromuscular performance among schoolchildren, as evidenced by reductions in maximum plantar pressure and improved weight-bearing symmetry under static, dynamic, and load-bearing conditions. These results suggest that targeted exercise interventions may be able to optimize load distribution and postural control in simple and complex motor tasks. The intervention produced consistent adaptations across all testing conditions, and greater improvements were observed as task demands increased. Such a pattern suggests that the exercise program improved both basic balance and postural stability and functional performance during more demanding motor tasks. Greater improvements were observed under dynamic and load-bearing conditions. More importantly, the increased weight-bearing symmetry further corroborates more bilateral coordination as well as optimal neuromuscular activation. From a biomechanical perspective, reductions in plantar pressures suggest more efficient transfer of force and decreased localized mechanical stress, crucial in mitigating overload-induced musculoskeletal abnormalities. These adaptations demonstrate the functional implications of structured neuromuscular training that encourage more economical movement patterns which enhance the overall quality of the movement in children. At a more practical level, results endorse the inclusion of structured exercise programs in school environments as a useful method to promote postural health, gait efficiency, and reducing the risk of musculoskeletal disorders in childhood. Overall, this study provides compelling evidence supporting the use of early, targeted PA interventions to promote healthy neuromuscular development and functional performance in pediatric populations.

### Study impact

Schoolchildren who participated in the 16-week structured exercise program showed improvements in plantar pressure distribution and weight-bearing symmetry across static, dynamic, and load-bearing conditions. These findings suggest favorable changes in load distribution and postural control; however, the present study was not designed to establish clinically meaningful thresholds. The results support further investigation of targeted neuromuscular training within school-based physical education programs as a potential approach to improve movement quality and reduce mechanical loading during functional tasks.

##  Supplemental Information

10.7717/peerj.21941/supp-1 Supplemental Information 1 Detailed 16-week structured exercise program

10.7717/peerj.21941/supp-2 Supplemental Information 2 Descriptive Statistics for Maximum Plantar Pressure during Standing (N/cm^2^)

10.7717/peerj.21941/supp-3 Supplemental Information 3 Descriptive Statistics for Maximum Plantar Pressure during Walking (N/cm^2^)

10.7717/peerj.21941/supp-4 Supplemental Information 4 Descriptive Statistics for Maximum Plantar Pressure during Load Condition (N/cm^2^)

10.7717/peerj.21941/supp-5 Supplemental Information 5 Descriptive Statistics for Weight-Bearing Symmetry (%)

10.7717/peerj.21941/supp-6 Supplemental Information 6 Comparison of plantar pressure during standing (boxplot) pre and post.”

10.7717/peerj.21941/supp-7 Supplemental Information 7 Pre-post comparison of plantar pressure during walking (boxplot)

10.7717/peerj.21941/supp-8 Supplemental Information 8 Plantar pressure versus load (bar plot) before and after training

10.7717/peerj.21941/supp-9 Supplemental Information 9 Raw data

## Additional Information and Declarations

### Data Availability

The following information was supplied regarding data availability: The raw measurements are available in the Supplemental Files.
