# Supplementary material for: Neuromuscular efficiency in schoolchildren: a 16-week structured exercise program improves plantar load distribution across static, dynamic and load-bearing tasks
Source: PeerJ. 2026 Nov 12;14:e21941. doi: 10.7717/peerj.21941 (PMC13770417; doi:10.7717/peerj.21941)
Supplement: Supplementary file 1 [file peerj-14-21941-s001.docx]

**Supplementary Table S1.** Detailed 16-week structured exercise program.

| **Weeks** | **Main objective** | **Exercises** | **Sets** | **Repetitions/Time** | **Progression** |
| --- | --- | --- | --- | --- | --- |
| 1–2 | Familiarization | Static balance, bridge, bird-dog | 2 | 10 reps / 20 s | Technique learning |
| 3–4 | Core activation | Plank, side plank, single-leg stance | 2–3 | 20–30 s | Increased holding time |
| 5–8 | Coordination | Tandem walking, cone drills, lunges | 3 | 10–12 reps | Greater movement complexity |
| 9–12 | Dynamic stability | Squats, hopping, agility ladder | 3 | 12–15 reps | Reduced base of support |
| 13–16 | Functional integration | Combined balance, gait, load-bearing drills (Schoolbag (≤10% body mass) | 3 | 15 reps | Increased task complexity |

*Exercise intensity and complexity were progressively increased according to participants' motor competence and ability to correctly perform each exercise. Whenever load-bearing exercises were performed, the external load (schoolbag) did not exceed 10% of the participant's body mass.*
