# Supplementary material for: Neuromuscular efficiency in schoolchildren: a 16-week structured exercise program improves plantar load distribution across static, dynamic and load-bearing tasks
Source: PeerJ. 2026 Nov 12;14:e21941. doi: 10.7717/peerj.21941 (PMC13770417; doi:10.7717/peerj.21941)
Supplement: Supplementary file 2 [file peerj-14-21941-s002.docx]

**Supplementary Table S2.** Descriptive Statistics for Maximum Plantar Pressure during Standing (N/cm²)

| **Group** | **Pre (M ± SD)** | **Post (M ± SD)** | **Δ (Post–Pre)** | **Cohen’s d** | **t(df)** | **p-value** |
| --- | --- | --- | --- | --- | --- | --- |
| Control (n = 73) | 12.71 ± 6.25 | 12.11 ± 6.42 | –0.60 | –0.10 | –0.84 (72) | .40 |
| Experimental (n = 74) | 13.50 ± 6.33 | 11.98 ± 6.90 | –1.52 | –0.27 | –2.31 (73) | .024* |

*Note. p < .05^*^ statistically significant*
