# Supplementary material for: Neuromuscular efficiency in schoolchildren: a 16-week structured exercise program improves plantar load distribution across static, dynamic and load-bearing tasks
Source: PeerJ. 2026 Nov 12;14:e21941. doi: 10.7717/peerj.21941 (PMC13770417; doi:10.7717/peerj.21941)
Supplement: Supplementary file 3 [file peerj-14-21941-s003.docx]

**Supplementary Table S3.** Descriptive Statistics for Maximum Plantar Pressure during Walking (N/cm²)

| **Group** | **Pre (M ± SD)** | **Post (M ± SD)** | **Δ (Post–Pre)** | **Cohen’s d** | **t(df)** | **p-value** |
| --- | --- | --- | --- | --- | --- | --- |
| Control (n = 73) | 51.67 ± 12.47 | 51.11 ± 12.79 | –0.56 | –0.08 | –0.68 (72) | .50 |
| Experimental (n = 74) | 52.41 ± 13.50 | 50.83 ± 14.11 | –1.58 | –0.28 | –2.45 (73) | .017* |

*Note. p < .05^*^ statistically significant*
