# Supplementary material for: Neuromuscular efficiency in schoolchildren: a 16-week structured exercise program improves plantar load distribution across static, dynamic and load-bearing tasks
Source: PeerJ. 2026 Nov 12;14:e21941. doi: 10.7717/peerj.21941 (PMC13770417; doi:10.7717/peerj.21941)
Supplement: Supplementary file 4 [file peerj-14-21941-s004.docx]

**Supplementary Table S4.** Descriptive Statistics for Maximum Plantar Pressure during Load Condition (N/cm²)

| **Group** | **Pre (M ± SD)** | **Post (M ± SD)** | **Δ (Post–Pre)** | **Cohen’s d** | **t(df)** | **p-value** |
| --- | --- | --- | --- | --- | --- | --- |
| Control (n = 73) | 57.09 ± 9.42 | 56.40 ± 9.67 | –0.69 | –0.11 | –0.91 (72) | .37 |
| Experimental (n = 74) | 54.18 ± 11.84 | 52.40 ± 12.41 | –1.78 | –0.31 | –2.63 (73) | .010* |

*Note. p < .05^*^ statistically significant*
