# Supplementary material for: Neuromuscular efficiency in schoolchildren: a 16-week structured exercise program improves plantar load distribution across static, dynamic and load-bearing tasks
Source: PeerJ. 2026 Nov 12;14:e21941. doi: 10.7717/peerj.21941 (PMC13770417; doi:10.7717/peerj.21941)
Supplement: Supplementary file 5 [file peerj-14-21941-s005.docx]

**Supplementary Table S5.** Descriptive Statistics for Weight-Bearing Symmetry (%)

| **Group** | **Pre (M ± SD)** | **Post (M ± SD)** | **Δ (Post–Pre)** | **Cohen’s d** | **t(df)** | **p-value** |
| --- | --- | --- | --- | --- | --- | --- |
| Control (n = 73) | 46.9 ± 3.7 | 47.6 ± 3.9 | +0.7 | 0.13 | 1.15 (72) | .25 |
| Experimental (n = 74) | 46.4 ± 4.1 | 49.2 ± 3.6 | +2.8 | 0.48 | 4.12 (73) | < .001** |

*Note. ** p < .001 statistically very significant.*
